# Electric stimulation-guided epidural analgesia for vaginal delivery: A randomized prospective study

**DOI:** 10.1371/journal.pone.0209967

**Published:** 2019-01-11

**Authors:** Chung Hun Lee, Sang Sik Choi, Mi Kyoung Lee, Jung Eun Kim, Dong Ik Chung, Mido Lee

**Affiliations:** Department of Anesthesiology and Pain medicine, Korea University Medical Center, Guro Hospital, Guro-Gu, Seoul, Republic of Korea; McGill University Health Centre, CANADA

## Abstract

**Background:**

The failure rate of epidural anesthesia using the loss of resistance technique is 13–23%.

**Objectives:**

To investigate the efficacy of epidural electric stimulation-guided epidural analgesia in vaginal delivery.

**Study design:**

An open label randomized prospective study.

**Methods:**

Laboring women were randomized to two groups: epidural catheter insertion using only a loss of resistance technique or a loss of resistance technique with confirmation by electric stimulation. Catheters in both groups were initially tested with 3 ml of 1% lidocaine and those with any evidence of motor blockade were considered intrathecal. Sensory blockade and an 11 point numerical rating score for pain were assessed 30 minutes after administration of an epidural bolus of 10 ml of 0.22% ropivacaine with fentanyl. Successful epidural analgesia was defined as a decrease of 2 or more in the pain score and a bilateral L1-T10 sensory blockade.

**Results:**

Thirty-one patients were randomized to each group. The first 20 patients in each group were enrolled in a pilot study and were also included in the final analysis. One patient in the electric stimulation group was excluded owing to dural puncture by the Tuohy needle. One patient in each group demonstrated motor blockade after test dose and were considered failures. The number (% (95% confidence interval)) of successful cases were 29 out of 30 (97% (85, 100%)) in the electric stimulation group and 24 out of 31 (77% (61, 89%)) in the loss of resistance group (*P* = 0.053). However, analysis of only patients with absence of motor blockade revealed that 29 out of 29 (100% (92, 100%)) patients in the electric stimulation group and 24 of 29 (80% (63, 91%)) patients in the loss of resistance group had adequate analgesia (*P* = 0.024).

**Conclusions:**

Although limited by lack of blinding, small study size and inclusion of pilot study data, this study suggests epidural electric stimulation improves the success rate of subsequent labor analgesia.

## Introduction

Epidural analgesia is an effective method to relieve pain associated with labor without impairing the motor nerve function. Moreover, the use of epidural analgesia during labor and the postpartum period has increased over time [1,2].

Loss of resistance to air or saline is the primary technique used to identify the epidural space [3], but it has a failure rate of 13% to 23% [4–8]. This may be because the loss of resistance technique is subjective, with the success rate dependent upon the practitioners’ skill and experience [4,5,6,9]. In addition, identification of the epidural space during labor becomes more difficult due to obesity, edema and the hormonally induced softening of the subcutaneous tissues and interspinous ligaments, which can result in a false positive loss of resistance [5,10,11]. Therefore, development of a simple, safe and objective technique to increase the success rate of epidural labor analgesia is an important goal.

A technique using electric stimulation to confirm the placement of a catheter in the epidural space has been reported. Tsui et al [12] identified the epidural space with electric stimulation using normal saline as a conductor. Electrical conduction can also be provided with a guidewire embedded in the epidural catheter. We wished to determine whether confirmation of an epidural catheter with electrical stimulation, after placement using the loss of resistance technique, could improve subsequent analgesia success rates in laboring women. In addition, we wished to evaluate the safety and efficacy of epidural catheter electrical stimulation in this population.

## Materials and methods

### Patient enrollment and study design

This randomized, prospective study was approved by the Institutional Review Board of the Korea University Medical Center, Guro Hospital, Seoul, Republic of Korea (MD14043) on March 11, 2015 and registered at Clinical-Trials.gov (NCT03161717) on May 18, 2017. The study was conducted between May 2017 and October 2017 after completion of a pilot study.

The study was explained to patients and written informed consent was obtained prior to enrollment. Patients in labor who were at 36 to 41 weeks’ gestation and admitted to the university hospital for vaginal delivery were included. All included patients were American Society of Anesthesiologists physical status I or II and had elected to receive epidural analgesia. Exclusion criteria were as follows: skin infection at injection site; difficult catheter placement owing to previous lumbar spinal surgery or deformity; presence of hemostatic disorder or use of antiplatelet therapy; injection of an analgesic within the previous 12 hours; or the presence of a cardiac pacemaker.

Sixty-two patients were randomized into two groups of 31 patients using a random numbers table prior to the start of epidural procedure. Group assignments were placed in sealed opaque envelopes which were opened at the time of patient enrolment.

Six experienced anesthesiology residents who had previously performed at least 30 epidural procedures with the loss of resistance technique placed all catheters. The residents were assigned to the study using another randomization table.

The patient’s blood pressure, heart rate, oxygen saturation and neurologic status were monitored for up to 72 hours after labor. For epidural catheter placement, patients were placed in the left lateral decubitus position. The site was aseptically prepared and the skin was infiltrated with 1% lidocaine. An 18-gauge Tuohy needle was inserted midline at the L3/4 or L4/5 interspinous space. The epidural space was then identified using loss of resistance to saline or air, at the discretion of the person performing the technique. In the loss of resistance only group, a 20-gauge closed tip and multi orifice epidural catheter (Perifix Soft Tip epidural anesthesia catheter; B. Braun, Germany) was then advanced through the Touhy needle. If the catheter could not be advanced, the Tuohy needle was repositioned and the epidural catheter was reinserted. In the electric stimulation group the epidural space was initially identified using the same technique as in the loss of resistance group, but a different catheter (20-gauge RegionalStim, open tip catheter, length: 800 mm; Sewoon Medical Co., Ltd, Seoul, Korea) was inserted. This epidural catheter has a built-in conductive guidewire (Nitinol, length; 1100 mm) with 800 mm inside the catheter and 300 mm exposed for connection to an electric nerve stimulator. The cathode of the electric nerve stimulator (Life-Tech EZstim, Stafford, TX, USA) was connected to the exposed guidewire and the anode was attached to an electrode on the patient’s calf. For stimulation, the current was gradually increased from 0 mA to 5 mA, with a frequency of 1 Hz and pulse-width of 300 ms. The minimum electric current that elicited an adequate motor response (paresthesia of a dermatome or motor response of a muscle group, including the hip adductors, iliopsoas, gluteus, or hamstrings) was recorded. In case of lack of adequate response at the maximum current the epidural catheter was adjusted; if an adequate response was still not achieved after catheter adjustment the Tuohy needle was repositioned and the epidural catheter was reinserted. Once an appropriate response was achieved, the guidewire was removed from the catheter.

In both groups the epidural catheter was advanced 4 cm beyond the tip of the Tuohy needle. After confirming negative aspiration of cerebrospinal fluid or blood, 3 mL of 1% lidocaine with 15 mcg of epinephrine (1:200000) was injected through the epidural catheter as a test dose. Motor testing was conducted by the physician who inserted the epidural catheter, before and 30 minutes after test dose injection. With the patient in the supine position, hip flexion, knee extension, knee flexion, ankle dorsiflexion, and plantar flexion were performed against manual resistance. The motor response was quantified using a manual muscle testing grading system with a scale from 0 (no muscle response) to 5 (normal muscle response).[13] If the motor testing grade after the test dose was 4 or less, motor weakness was diagnosed, the epidural catheter was considered as possibly being intrathecal and the case was treated as a failure.

If motor blockade was absent 30 minutes after injection of the test dose, the patient’s pain was assessed using an 11-point verbal numeric rating scale (NRS), with 0 indicating no pain and 10 indicating unbearable pain. If patients had an NRS score of 3 or more they were given an epidural bolus. If a patient had an NRS score less than 3 they were monitored until their NRS score reached 3 and then given an epidural bolus. The bolus injection contained 50 mcg of fentanyl, 3 mL of 0.75% ropivacaine and 6 mL of normal saline (0.225% ropivacaine; total volume: 10 mL). Further analgesia was provided with a 3 to 10 mL/hour continuous epidural infusion of a solution containing 75 mcg of fentanyl, 8.5 mL of 0.75% ropivacaine, and 40 mL of normal saline (0.1275% ropivacaine; total volume: 50 mL), titrated to relieve the patient’s pain.

### Data collection

Baseline demographic characteristics were recorded for all patients. Pain was assessed using the NRS at the time points of pre-epidural catheterization, pre-epidural bolus injection and 30 minutes post-epidural bolus injection. The sensory blockade was assessed 30 minutes after epidural bolus by the anesthesiologist who performed the bolus injection, using needle prick or alcohol swab. The difference between the pre- and post-epidural bolus NRS scores were calculated to assess the efficacy of epidural analgesia. Successful epidural analgesia was defined as the presence of adequate sensory block (from the L1 to the T10 dermatome, bilaterally) and a 2 or more decrease in NRS pain score after initial bolus through the epidural catheter. Failure of epidural analgesia was defined as the presence of one of the following: significant motor block after test dose injection, lack of sensory block, or less than a 2-point difference in NRS score after delivering the initial bolus through the epidural catheter. Patient satisfaction was evaluated via postpartum interview and was graded by the patients on a scale of 1 to 5, where 1 represented very unsatisfied; 2, slightly unsatisfied; 3, moderately satisfied; 4, slightly satisfied; 5, very satisfied. To assess the effect of epidural electric stimulation on the neonate, Apgar scores at 1- and 5-minutes were compared between the groups. The additional time required for epidural electric stimulation was determined by the difference (in seconds) from the loss of resistance to the confirmation of the epidural space through electric stimulation. The additional time included the time needed to redirect the needle and catheter. Patients for whom an epidural catheter could not be placed due to procedure-related complications, such as a dural puncture by the Tuohy needle, were excluded from the study. In this study, blinding of both the patient and care providers was not possible; moreover, the observer evaluating the patient could not be blinded. Thus, this was a non-blinded study.

### Statistical analysis

The success rate was compared between groups using the Fisher’s exact test. All variables were analyzed using the Kolmogorov-Smirnov test to assess the normality of data distribution. Age, weight, body mass index, and gestational age were normally distributed. Maternal satisfaction, baseline NRS score, NRS difference, and neonatal Apgar scores were not normally distributed and were analyzed using the Mann-Whitney U test. A two-sided P value < 0.05 was considered statistically significant. Data are presented as mean ± standard deviation or median [interquartile range]. Data were analyzed using Statistical Package for the Social Sciences (SPSS) software (version 17.0; SPSS 157 Inc., Chicago, IL, USA).

#### Sample size

Initial review of the literature retrieved no appropriate reference for estimation of success rates with epidural electric stimulation in this population. Therefore, the sample size calculation was performed after completion of the pilot study and before the main study. G*Power software (version 3.1.9.2) was used to determine the number of patients needed per group, using the z test with the p1 proportion of 0.99, p2 proportion of 0.77, error probability of 0.05 and power value of 0.8. The p2 proportion was set at 0.77 based on a maximum failure rate of epidural anesthesia of 23% reported in a previous paper [6]. Based on these criteria, we determined that there needed to be at least 26 patients in each group. Considering the drop-out rate, it was judged that more patient data than those of minimum sample size were required to obtain significant differences. Therefore, including the patients in the pilot study, we enrolled 31 patients in the loss of resistance group and 31 patients in the electric stimulation group.

## Results

**Fig 1:**
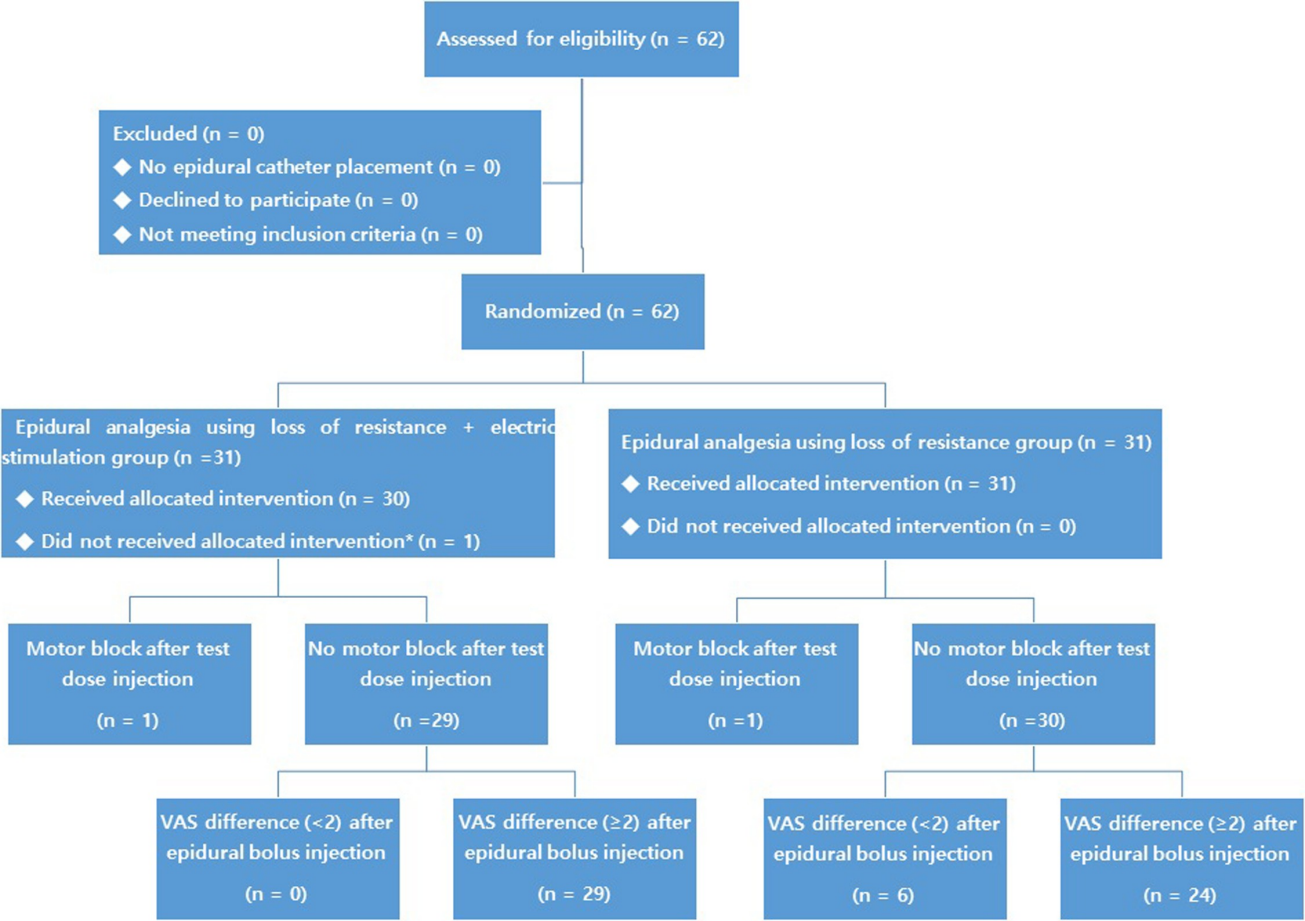
Flow diagram of patients. *Excluded due to dural puncture by Tuohy needle.

In total 62 patients were enrolled, with 31 randomized to each group. Six anesthesiology residents were randomly assigned to the patients, with each performing the same number of epidurals in both groups. All patients (n = 31) received the intended therapy in the loss of resistance group, but one patient was excluded before catheter placement in the electrical stimulation group (n = 30) due to dural puncture with the Tuohy needle. All other patients were included in the final analysis (Fig 1).

There were no significant group-wise differences in the baseline characteristics (Table 1).

**Table 1:** Baseline characteristics of the patients.

	Electric stimulation group	Loss of resistance group
	(n = 30)	(n = 31)
**Age (years)**	31.8 ± 4.9	33.2 ± 4.5
**Weight (kg)**	66.5 ± 11.0	68.4 ± 7.7
**Body mass index (kg/m**^**2**^**)**	25.5 ± 4.0	26.1 ± 3.4
**Gestational Age (weeks)**	38.5 ± 1.9	39.0 ± 1.8
**Baseline NRS**	6 [6–7]	7 [6–7]
**Cervical dilatation (cm)**	5 [4–5]	5 [4–5]
**Parity of the patient**	0 [0–0.5]	0 [0]
**Patients undergoing induction**	29 (97% (85, 100%))	29 (94% (81, 99%))
**Patients treated with oxytocin**	22 (73% (56, 87))	23 (74% (57, 87%))
**Patients whose epidural bolus was delayed due to pain score < 3**	14 (47% (30, 64%))	12 (39% (23, 56%))

NRS: numerical rating scale of 0 to 10. Data are presented as mean ± standard deviation, median [interquartile range] or number (% (95% confidence interval)).

Epidural analgesia was successfully achieved in 24 of 31 patients (77%; 95% confidence interval: 61, 89%) in the loss of resistance group and 29 of 30 patients (97%; 95% confidence interval: 85, 100%) in the electric stimulation group (*P* = 0.053) (Table 2).

**Table 2:** Clinical outcomes in the epidural electric stimulation and loss of resistance groups.

	Electric stimulation group	Loss of resistance group	*P* value
	(n = 30)	(n = 31)	
**Successful analgesia**	29 (97% (85, 100%))	24 (77% (61, 89%))	0.053
**Reason for failed epidural analgesia**			
** Inadequate sensory blockade**	0 (0% (0, 8%))	6 (19% (9, 36%))	0.024
** Patients with motor blockade**	1 (3% (0, 15%))	1 (3% (0, 14%))	1.0
** Patients without reduced NRS by at least 2 points**	0 (0% (0, 8%))	6 (19% (9, 36%))	0.024
**Maternal satisfaction**	5 [4–5]	4 [4–5]	0.11
**Apgar score (1 min)**	9 [8–10]	9 [8–10]	0.85
**Apgar score (5 min)**	10 [9–10]	10 [9–10]	0.94
**NRS difference**	4 [4–4]	4 [3–5]	0.30

NRS: numerical rating scale of 0 to 10. Values are presented as median [interquartile range] or number (%, (95% confidence interval)). Success rate was analyzed using the Fisher’s exact test. Maternal satisfaction, Apgar scores, and difference in NRS were analyzed using the Mann-Whitney U test.

One patient in each group showed decreased motor function after the test dose injection and both were considered as failures due to possible intrathecal catheter placement. However, neither of these patients experienced complications such as post-dural puncture headache. In a subgroup analysis of only the patients without motor blockade, 29 (100%, 95% confidence interval: 92, 100%) patients in the electric stimulation group and 24 (80%, 95% confidence interval: 63, 91%) patients in the loss of resistance group showed adequate analgesia and sensory blockade (*P* = 0.024). After a single bolus injection of epidural medication, the NRS score decreased by a median [interquartile range] of 4 [3–5] points in the loss of resistance group and 4 [4–4] in the electric stimulation group (*P* = 0.3).

There were no differences noted in patient satisfaction, NRS difference before and after epidural bolus, procedure-related complications, and Apgar scores between the two groups (Table 2).

**Fig 2:**
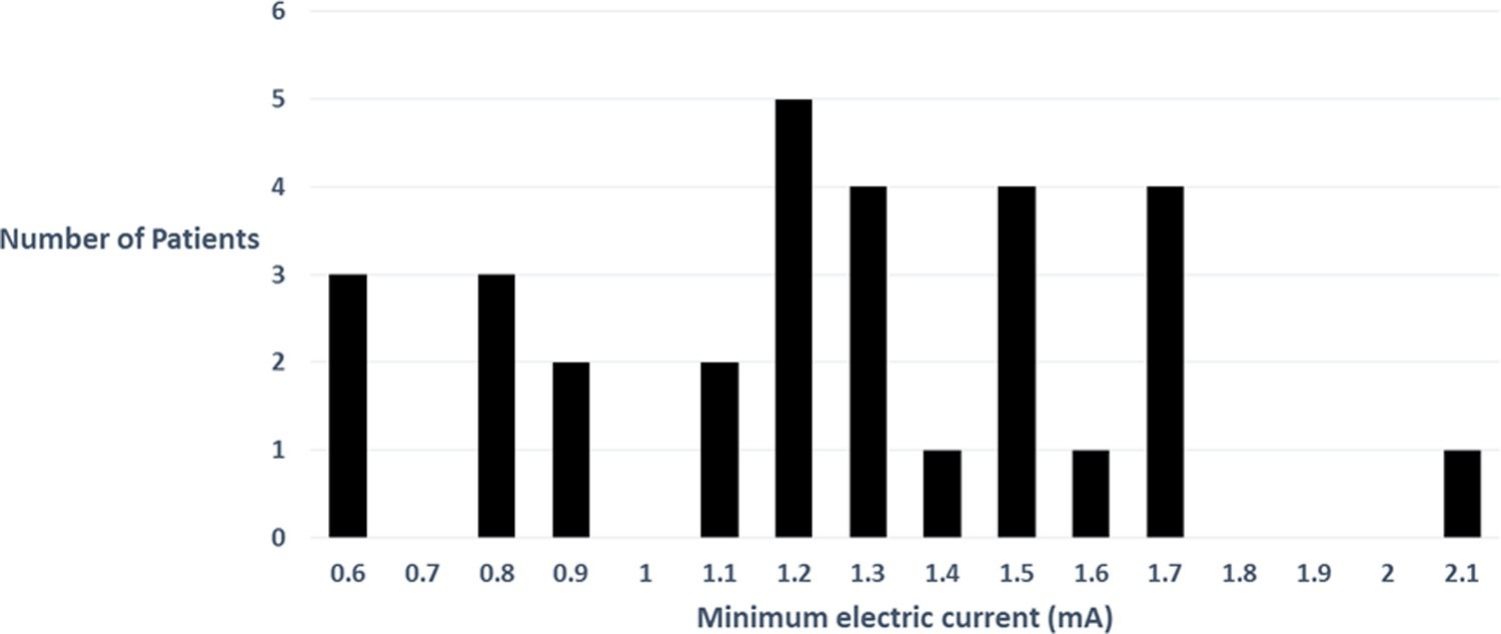
Minimum electric current for evoking paresthesia or muscle contraction in the electric stimulation group (n = 30). For confirming epidural catheter placement the minimum current required was 0.6–2.1 mA and the median [interquartile range] current was 1.25 [0.9–1.5] mA (frequency: 1 Hz; pulse-width: 300 ms).

In the electric stimulation group the minimum electric current ranged from 0.6 to 2.1 mA (Fig 2). The minimum current in the patient with evidence of motor blockade after the test dose injection was 0.6 mA. Five of the patients in the electric stimulation group demonstrated no response to electrical stimulation, despite increasing the current to 5 mA. These catheters were repositioned or the epidural space was relocated and an adequate response was demonstrated. The mean (± standard deviation) and median [interquartile range] additional time from observation of loss of resistance to confirmation of the epidural space by electric stimulation was 150 ± 90 seconds and 120 [90–150] seconds respectively. All patients achieved full recovery without evidence of neurologic and cardiovascular side effects.

## Discussion

This study compared labor analgesia success rates in patients who had an epidural catheter placed using a traditional loss of resistance technique to patients whose catheter was placed with the loss of resistance technique and then confirmed with electrical stimulation. The failure rate with only the use of loss of resistance was 23%, which agrees with published rates [4–8]. In the electric stimulation group 16% of catheters were repositioned after lack of appropriate electrical response, resulting in a 97% final success rate. If it is assumed that these catheters would not have provided successful analgesia, the failure rate between groups would have been similar. This suggests that electrical stimulation may have prevented a large percentage of failed labor analgesia, although it is not known how the catheters would have performed if they weren’t repositioned. In any case, the final difference in failure rates between groups does provide some evidence that electric stimulation may improve epidural catheter success. However, there are many limitations to this study that weaken its ability to convincingly prove that epidural electric stimulation is useful in these patients.

First, analgesia may vary due to differences in the catheters used in each group. Previous reports [14,15] have shown that a closed tip catheter is more effective for sensory block than an open tip catheter. The Perifix soft catheter used in the loss of resistance group is a closed type multi orifice catheter that should yield better results when compared to the RegionalStim end-hole catheter used in the electric stimulation group. However, in this study, a higher success rate was achieved using an end-hole catheter. It is possible that confirming the epidural space by electric stimulation offsets the difference in analgesic effect based on catheter type.

Another limitation of this present study is that it was performed in a university hospital where anesthesiology residents performed epidural analgesia. Therefore, the failure rate of epidural analgesia with the conventional loss of resistance technique was high. The epidural electric stimulation technique may not be very useful for experienced practitioners accustomed to a relatively high success rate. However, even for experienced practitioners, identifying the epidural space by electric stimulation might increase the success rate of epidural analgesia, especially in patients with difficult epidural catheterization. It is also important to note that this study had an open-label design, with the inherent risk of bias favoring the stimulation group. The sample size of this study may be inappropriate due to a post-hoc power calculation. Additionally, no adjustment was performed for the interim analysis of the pilot study patients who were included in the final analysis. This also increases the risk of falsely finding a significant effect for the stimulation group. Therefore, blinded studies with larger sample sizes, performed by practitioners with more experience, are needed to validate our findings.

We found very little difference between groups in terms of secondary outcomes. There was no statistically significant difference in maternal satisfaction and NRS decrease between the two groups. Epidural electric stimulation with low current is unlikely to affect the fetus or newborn; nevertheless, we evaluated Apgar scores to confirm the safety of the procedure and found no differences in the 1- or 5- minute Apgar scores. We must note that this study may have lacked the power to analyze these secondary outcomes effectively.

Tsui et al. [12] introduced the use of electric stimulation to confirm catheter placement in the epidural space. Compared with the standard test dose, they reported a sensitivity of 100% and a specificity of 91.6% for epidural stimulation. Subsequently, several investigators have developed electric stimulation methods to confirm the correct placement of catheters in the epidural space, and have reported no side effects [16–18]. Previous studies have used an epidural catheter with a fixed electrode at the distal tip with the electric impulse conducted through normal saline within the lumen of the catheter [12,16]. This method requires a relatively high electric current (up to 14 mA) to stimulate the epidural neural structures. In our study we used a conductive guidewire to transmit electric current accurately and effectively into the epidural space.

Tsui et al. [12] also reported that motor responses elicited by electric stimulation (current range, 1–10 mA; frequency, 1 Hz; pulse-width, 200 ms) confirmed the placement of the catheter within the epidural space. A motor response to less than 1 mA stimulation was considered to indicate the misplacement of catheter within the intrathecal space [12]. In a study aimed at determining the current required to confirm catheter placement in the epidural and intrathecal spaces, Sutherland et al. [16] reported that the mean current required to produce appropriate muscle contraction was 7.8 ± 3.3 mA (range, 2–14 mA; frequency, 2 Hz; pulse-width, 200 ms) in the epidural space and 1.3 ± 0.8 mA (range, 0.05–2.4 mA; frequency, 2 Hz; pulse-width, 200 ms) in the intrathecal space; the authors concluded that in an epidural stimulation test, the electric current required for intrathecal catheter placement was lower than that required for epidural catheter placement. In the present study, the minimum current for evoking segmental paresthesia or a motor response was between 0.6 mA and 2.1 mA (frequency, 1 Hz; pulse-width, 300 ms). Twenty-nine patients underwent successful epidural analgesia in the electric stimulation group, of which seven patients showed partial sensation or motor reaction at < 1 mA. This suggests that a neural response to < 1 mA stimulation when using a catheter with conductive guidewire does not necessarily indicate that the catheter is in the intrathecal space.

Although this study does provide some evidence that epidural electric stimulation improves the success rate of catheter placement for labor analgesia, its limitations mandate caution. Blinded studies including larger sample sizes performed by practitioners with more experience are needed to further validate this hypothesis and to assess the effectiveness of routine use of this technique in clinical practice.

## Supporting information

S1 File A CONSORT checklist for the present study. (DOC)

S2 File A Dataset for the present study. (XLSX)

S3 File A CONSORT flow diagram. (DOCX)

S4 File IRB (English). (PDF)

S5 File IRB (Original). (PDF)

S6 File A Trial study protocol. (PDF)
