# Supplementary material for: Electric stimulation-guided epidural analgesia for vaginal delivery: A randomized prospective study
Source: PLoS One. 2019 Jan 11;14(1):e0209967. doi: 10.1371/journal.pone.0209967 (PMC6329494; doi:10.1371/journal.pone.0209967)
Supplement: S3 File — (DOCX) [file pone.0209967.s003.docx]

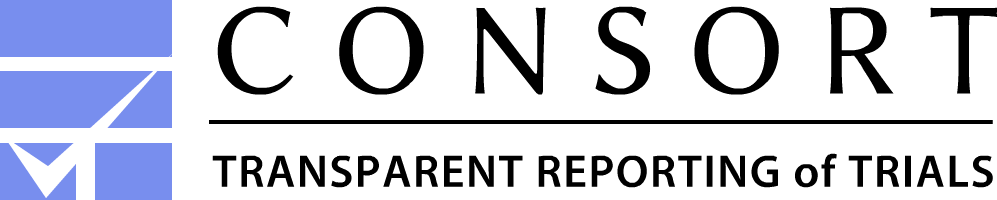


**CONSORT 2010 Flow Diagram**

Analysed (n = 30)
♦ Excluded from analysis (give reasons) (n = 0)

Allocated to intervention (n = 31)

♦ Received allocated intervention (n = 31)

♦ Did not receive allocated intervention (give reasons) (n = 0)

Allocated to intervention (n = 31)

♦ Received allocated intervention (n = 30)

♦ Did not receive allocated intervention (give reasons) (n = 1)

Lost to follow-up (give reasons) (n= 0)

Discontinued intervention (give reasons) (n = 0)

Lost to follow-up (give reasons) (n = 0)

Discontinued intervention (give reasons) (n = 0)

Randomized (n = 62)

Excluded (n = 0)

♦  Not meeting inclusion criteria (n = 0)

♦  Declined to participate (n = 0)

♦  Other reasons (n = 0)

## Follow-Up

## Analysis

Analysed (n = 31)
♦ Excluded from analysis (give reasons) (n = 0)

## Enrollment

## Allocation

Assessed for eligibility (n = 62)
