# Supplementary material for: Electric stimulation-guided epidural analgesia for vaginal delivery: A randomized prospective study
Source: PLoS One. 2019 Jan 11;14(1):e0209967. doi: 10.1371/journal.pone.0209967 (PMC6329494; doi:10.1371/journal.pone.0209967)
Supplement: S4 File — (PDF) [file pone.0209967.s004.pdf]

고려대학교구로병원 KOREA UNIVERSITY GURO HOSPITAL	**Institutional Review Board**	**연구계획서**

1. Title and steps of clinical trials

A comparative study of success rate, efficacy, safety between electric stimulation-guided epidural catheter(RegionalStim®) placement and the loss of resistance conventional method for vaginal delivery (by pilot study)

2. Name and address of clinical trial institution

Department of Anesthesiology and Pain medicine, Korea University Medical Center, Guro Hospital

Gurodong Road 148, Guro-Gu, Seoul, Republic of Korea

3. Name and title of the lead researcher, co-researcher

Lead researcher: Sang Sik Choi

Co-researcher: Chung Hun Lee

Co-researcher: Mi Kyoung Lee

Co-researcher: Jung En Kim

4. Name and job title of medical device manager

Lead researcher: Sang Sik Choi

Co-researcher: Chung Hun Lee

5. Name and address of clinical trial client

None

6. Background and purpose of clinical trial

1) Study purpose

Epidural analgesia is an effective method to relieve pain associated with labor without blocking motor nerve function. The use of epidural analgesia as a method of pain relief during labor and in post-partum period has increased. (1)

Currently, the loss of resistance (LOR) technique is used to identify the epidural space.

However, the failure rate of epidural anesthesia using the LOR technique is 10% to 27%. (2-3)

Therefore, to increase the success rate of epidural analgesia, a simple, safe, and objective technique to confirm correct placement of the epidural catheter in the epidural space would be useful.

To identify the epidural space, we will use electric stimulation and a conductive guidewire embedded in the catheter. The guidewire serve as an electric conductor. We will investigate whether epidural electric stimulation improve epidural analgesia in patients in labor. We will evaluate the efficacy and safety of electric stimulation-guided epidural analgesia for vaginal delivery.

2) Present status of domestic and foreign researches and related research

1. Tsui BC, Tarkkila P, Gupta S, Kearney R. Confirmation of caudal needle placement using nerve stimulation. Anesthesiology 1999;91:374-378. (4)

2. Tsui BC, Gupta S, Finucane B. Confirmation of epidural catheter placement using nerve stimulation. Canadian journal of anaesthesia = Journal canadien d'anesthesie 1998;45:640-644. (5)

3) For clinical trials, the results of the previous phase clinical trials

I mentioned in item 1.

7. The name of the product (or the generic name of the main ingredient), the raw material and its quantity, shape, etc. (if applicable)

RegionalStim® (Neuroplasty Cathter Set)

8. Target disease (indications)

Normal delivery patients who wants only painless delivery

9. Subject selection criteria and exclusion criteria

1) Selection Criteria

① Normal delivery patients who wants only painless delivery

① Normal delivery patients who submitted written consent

2) Exclusion Criteria

① If there is or is a history of cancer or neurological, psychiatric, heart, liver, hematologic, muscular, skin, genital or immunodeficiency disorders

① If there is skin disease at the site where epidural anesthesia should be performed

① If you have a history of recent medical treatment / surgery

① In cases where the catheter is difficult to move or place in the epidural space due to spinal surgery

① If you have blood clotting disorder or are receiving anticoagulant treatment

① Patients with medical devices due to neuropathic pain (spinal cord stimulator, intrathecal pump or peripheral nerve stimulator)

① Other, if the researcher finds it inappropriate to participate in clinical trials

10. Number of target subjects and calculation basis

A reference to the success rate for epidural anesthesia is currently reported, but we believe that this success rate varies greatly depending on the proficiency of the practicing physician. In this study,

we compared the results of epidural anesthesia (loss of resistance technique only) and epidural anesthesia with epidural electric stimulation method in anesthesiology residents, who had previously performed at least 30 epidural procedures with the LOR technique. The pilot study will be used for comparative study. The target number of pilot study will be 20 patients per group.

After the pilot study, we plan to continue the study by calculating the number of subjects required for the clinical trial based on the clinical scores before the clinical trial

11. Period of clinical trial

6 months after IRB approval

12. Clinical test method (including usage amount, use method, period of use, combination therapy etc.)

The authors used an LOR technique to mount an epidural catheter and use RegionalStim® to check if the catheter is properly positioned in the epidural space by electric stimulation.

Placement of the epidural catheter, electric stimulation, and whether the stimulation has occurred should be done in the following order.

① Patient was placed in the lateral decubitus position and L3/4 or L4/5 interspinous space is defined as puncture site.

② Make an antiseptic skin preparation around the puncture site.

③ After the skin anesthesia is performed using a local anesthetic agent in the puncture site, the Tuohy needle inserts midline at the L3/4 or L4/5 interspinous space. The LOR technique is performed until the epidural space is reached, and the identification of epidural space is confirmed by loss of resistance.

④ After reaching the epidural space, insert a RegionalStim® catheter.

⑤ Place the RegionalStim® catheter up 5 cm

⑥ When the RegionalStim® catheter is placed at the desired position, the electric stimulator and the built-in guide wire are connected through the connector, starting from the low stimulus and increasing to the stimulation intensity that the patient starts to feel the stimulus. At this time, the intensity of the stimulus felt first is recorded, and the region where the stimulus is felt is described in detail. This confirms the location of the appropriate stimulus.

⑦ The appropriate concentration and volume of local anesthetic is applied, and after 10 minutes, motor, sensory block, and strength are checked.

13. Observation items, clinical examination items and observational examination methods

1) Primary endpoint

Success rate and complications of epidural anesthesia in conventional LOR technique and RegionalStim®

2) Secondary endpoint

Sensory, motor block and strength after epidural anesthesia, adequacy of anesthesia

The time taken to perform epidural anesthesia, ease of procedure

Patient satisfaction

14. Predicted Side Effects and Precautions for Use

There are no major adverse events that may occur with the existing epidural anesthesia (described below).

1) Side effects that may occur during epidural anesthesia are allergic reactions caused by disinfectants and local anesthetics. Very rarely, local anesthetics may inadvertently enter the blood vessels, which can cause neurotoxic reactions (dizziness, vomiting, convulsions, etc.), nerve damage, and nerve paralysis. Unintentional epidural puncture can also occur and this can lead to

headache. If symptoms are severe, epidural blood patch may be necessary.

2) There are rare side effects that may occur after epidural anesthesia. However, injection site epidural hemorrhage may cause hematomas around the nerve, or when infected, it may compress or irritate the nerve. In this case, abnormal sensation, nerve damage, Symptoms may occur. Surgical treatment may be necessary if the symptoms are severe.

15. Research suspension and dropout criteria

1) If the subject withdrew his / her consent to participate in the clinical trial

2) If the test can not be continued due to a serious adverse reaction or sudden accident

3) If the subject is unable or unwilling to comply with the visits and procedures specified in the plan

4) If the test is difficult due to the manifestation of severe complications

5) Subjects who do not meet the selection criteria participate in the test

6) If the subject who falls under the exclusion criteria participates in the examination

7) Others, if the clinical investigator decides that the test will interfere

16. Evaluation Criteria of Validity, Evaluation Method and Analysis Method (Statistical Analysis Method)

1) Primary efficacy end point, secondary efficacy end point

① Primary efficacy evaluation variable

; Success rate of epidural anesthesia in conventional LOR technique and electric stimulation

① Secondary efficacy evaluation variable

; Sensory, motor block and strength after epidural anesthesia, adequacy of anesthesia

The time taken to perform epidural anesthesia, ease of procedure

Patient satisfaction

2) Validity evaluation method and analysis method

The pupose of this study was to identify the epidural space by electric stimulation using RegionalStim® and compare it with the conventional LOR method. We will investigate the success rate of epidural anesthesia performed by both methods, and we want to confirm the difference in the degree of anesthesia strength and pain control according to the position of the catheter. We compare the location of the stimulation that occurred when the catheter was inserted and the position of the motor, sensory block, and the degree of epidural anesthesia through the catheter, and whether the appropriate anesthesia was performed during delivery.

17. Criteria, evaluation methods and reporting methods of safety including side effects

Most of the side effects that can occur in this study are side effects of epidural anesthesia, and other side effects are unlikely to occur. Epidural anesthesia is widely used as a method of local anesthesia for painless delivery and is an anesthetic method that is prepared for treatment methods and treatment in the event of side effects. We will record the adverse reactions occurring through the case report, and we will do our best to treat and compensate the victim as well as report side effects when serious side effects occur.

18. Research Schedule

	Jan	Feb	Mar	Apr	May	Jun	Jul	Aug	Sep	Oct	Nov	Dec
Pilot study		⦿	⦿	⦿	⦿	⦿						
Calculate the number of subjects		⦿	⦿	⦿	⦿	⦿						
Subject clinical trial			⦿	⦿	⦿	⦿	⦿	⦿	⦿	⦿		

Data collection and statistical analysis										◉	◉	
Results analysis and dissertation writing										◉	◉	◉
Report clinical trial results												◉

19. Matters concerning the treatment of subjects after clinical trials

After the clinical trial, the patient will be monitored for signs of vital signs and side effects after delivery, as well as for the normal delivery patients who have not participated in clinical trials.

20. Measures to protect the safety of subjects

The purpose of this study was to investigate the ethical and legal requirements of the study, and to conduct clinical trials in accordance with the approved protocol, We will obey the fundamental spirit of the Declaration. If there is a violation of human rights on the subject during this study, the clinical trial ethics review committee will be notified.

Information identifying the subject's identity will be kept confidential by the researcher, and the study material will be recorded as initial and coded subject identification information. In addition, subject and research information will be stored on a computer with limited access, and the research will be described and agreed in a separate space to protect the subject's identity. Even when the results of clinical trials are published, the information will remain confidential.

21. What else is needed to conduct clinical trials safely

None

22. References

1. Davies SJ, Paech MJ, Welch H et al. Maternal experience during epidural or combined spinal-epiduaral anestehsia for cesarean section: A prospective and randomized trial anesth analg 1997;85:607-613

2. Alahuhta S, Kangas-Saarela T, Hollmen AI, Edstrom HH. Visceral pain during caesarean section under spinal and epidural anesthesia with bupivacaine. Act anaesthesiologica Scandivavica 1990;34:95-98

3. Rawal N, Schollin J, Wesstrom G. Epidural versus spinal combined epidural block for cesarean section. Act anaesthesiologica Scandivavica 1988;62:31-66

4. Tsui BC, Tarkkila P, Gupta S, Kearney R. Confirmation of caudal needle placement using nerve stimulation. Anesthesiology 1999;91:374-378.

5. Tsui BC, Gupta S, Finucane B. Confirmation of epidural catheter placement using nerve stimulation. Canadian journal of anaesthesia = Journal canadien d'anesthesie 1998;45:640-644.

23. Subject consent form and statement form (attached)

Attached.

24. Covenant on Victims Compensation (Attachment)

Attached.

25. Case report (CRF) form (attached)

Attached.
