# Supplementary material for: Electric stimulation-guided epidural analgesia for vaginal delivery: A randomized prospective study
Source: PLoS One. 2019 Jan 11;14(1):e0209967. doi: 10.1371/journal.pone.0209967 (PMC6329494; doi:10.1371/journal.pone.0209967)
Supplement: S5 File — (PDF) [file pone.0209967.s005.pdf]

[서식04]

고려대학교구로병원 KOREA UNIVERSITY GURO HOSPITAL	Institutional Review Board	연구계획서

※다음의 항목으로 기재하시고 해당사항이 없는 항목에는 '해당사항 없음'으로 표시하시기 바랍니다.
※하단에 연구계획서의 version을 명시해 주시기 바랍니다.

1. 임상시험의 명칭 및 단계(해당되는 경우)
   자연분만 시 무통분만을 위한 경막외 마취시 기존의 저항소실법만을 이용한 경막외 마취와 저항소실법과 RegionalStim®을 이용한 경막외 마취의 효과 및 성공률에 대한 비교연구(by pilot study)

2. 임상시험 실시기관명 및 주소
   고려대학교 구로병원 통증클리닉
   서울특별시 구로구 구로동 80번지 고려대학교 구로병원 통증클리닉

3. 임상시험 책임연구자, 담당자, 공동연구자의 성명 및 직명
   책임연구자: 최상식 (부교수)
   공동연구자: 이충훈 (전공의)
   공동연구자: 이미경 (교수)
   공동연구자: 김정은 (임상조교수)

4. 의료기기관리자의 성명 및 직명(해당되는 경우)
   책임연구자: 최상식 (부교수)
   공동연구자: 이충훈 (전공의)

5. 임상시험 의뢰자명 및 주소
   해당사항 없음

6. 임상시험의 배경 및 목적
   1) 연구 목적
   자연분만 시 무통분만을 위한 경막외 마취는 흔히 사용되는 마취방법으로 자연분만 전,후의 통증관리에 유용한 부위마취 방법입니다. 그리고 경막외 마취의 대다수의 문헌보고는 긍정적인 결과를 보였습니다.(1)하지만 경막외 마취의 실패율은 20% ~ 40%로 높이 보고되고 있는 실정입니다.(2-3) 실패의 흔한 원인은 경막외 카테터가 올바른 위치에 거치되지 못하는 것으로 기존에 거치 방법으로 사용하고 있는 loss of resistance(LOR) 으로는 올바른 카테터 위치를 보장하지 못하는 것이 사실입니다. 이에 본 연구는 blind로 기존의 LOR을 이용한 경막외 마취와 RegionalStim을 이용하여 전기자극을 통해 카테터 위치를 확인하는 두가지 방법을 비교 연구하여 성공률을 비교해 볼 것이며 올바르게 카테터가 위치 해 있는 경우에 한하여 경막외 마취의 적절성 및 자연분만 전,후에 적절한 통증 조절이 되었는지에 대하여 연구할 계획입니다.

   2) 국내외 연구 현황 및 해당 연구와 관련된 선행 연구 결과
      1. 소아에서 caudal epidural block 시 전기자극을 주어 위치를 확인하는 임상시험이 있으며

predictive value가 100%로 맹목적으로 시행한 것보다 높게 나온 연구가 있습니다.(4)

2. 경막외 카테터 거치시 신경자극기를 이용한 연구가 있습니다. 이 역시 민감도, 특이도가 기존 방법보다 높게 나타났습니다.(5)

3) 임상시험의 경우 선행단계 임상시험의 성적(임상례수-계획된 수, 실제 대상 수, 완료된 수, 중도 탈락자 수 및 이유, 부작용 등에 대한 사항 포함)

1번 항목에서 언급하였습니다.

7. 의료기기의 상품명(또는 주성분의 일반명), 원자재 및 그 분량, 형상 등(해당되는 경우)

RegionalStim® (Neuroplasty Cathter Set)

8. 대상질환(적응증)

무통분만을 원하는 자연분만 산모

9. 피험자의 선정기준, 제외기준

1) 선정기준

① 무통분만을 원하는 자연분만 산모

① 서면동의서를 제출한 산모

2) 제외기준

① 시험 결과의 해석, 안전성, 피험자 참여에 영향을 줄 수 있는 암 또는 신경학적, 정신과적, 심장, 간, 혈액학적, 근육, 피부, 생식기, 면역결핍 장애의 병력이 있거나 현재 있는 경우

① 경막외 마취를 시행해야 하는 부위의 피부질환이 있는 경우

① 최근에 중요한 의학적 처치/수술을 받았던 과거력이 있는 경우

① 척추수술로 인해 경막외 공간에서의 카테터 진행이나 거치가 어려운 경우

① 혈액응고장애가 있거나 항응고제 치료를 받고 있는 경우

① 신경병증성 통증으로 인해 의료기기를 적용중인 환자 (spinal cord stimulator, intrathecal pump 또는 peripheral nerve stimulator)

① 기타, 연구자가 임상시험 참여에 부적합하다고 판단한 경우

10. 목표 피험자의 수 및 산출 근거

경막외마취(저항소실법 만을 이용한 방법)에 대한 성공률 및 분만 후 통증 조절에 대한 reference는 현재 보고되어 있으나 이 성공률 및 분만 후 통증 조절 정도는 시행하는 의사의 숙련도에 의해 크게 차이가 난다고 생각했습니다. 그래서 본 저자들은 마취통증의학과 전공의 2년차, 3년차 각각 두 명이 시행하는 경막외 마취(저항소실법 만을 이용한 방법)과 저항소실법에 RegionalStim 방법을 추가한 경막외 마취의 성공률 및 분만 후 통증 조절 정도에 대해 우선 pilot study를 통해 비교연구하기로 하였고 Pilot study의 목표 피험자수는 약 군당 20명으로 잡고 연구할 계획입니다. Pilot study가 끝나고 해당 임상시험 전 임상성적을 토대로 임상시험에 필요한 피험자 수를 산출 하여 추가적으로 연구를 계속 진행할 계획입니다

11. 임상시험 기간

IRB 승인 후 6개월 동안

12. 임상시험 방법(사용량ㆍ사용방법ㆍ사용기간ㆍ병용요법 등을 포함한다)

저자들은 LOR technique을 사용하여 경막외 카테터를 거치시키고 RegionalStim® 을 이용하여 전기적 자극을 통해 경막외공간에 카테터가 제대로 위치해 있는지 확인해보려고 합니다.

경막외 카테터 거치 및 전기자극, 자극발생 여부 확인은 다음 순서로 한다.

① 환자를 측와위로 눕히고 L3/4 사이를 천자부위로 정한다.
② 천자부위를 중심으로 시술이 이루어지는 부위를 antiseptic skin preparation 한다.
③ 천자부위에 국소마취제를 사용해 피부마취를 시행한 후 경막외 천자바늘을 척추체에 따라 midline approach를 이용하여 경막외 공간에 접근한다. 경막외 공간에 도달할 때까지 LOR technique을 시행하고, 경막외 공간에 도달 여부는 저항 소실 양상으로 확인한다.
④ 경막외 공간에 도달하면 RegionalStim 카테터를 삽입한다.
⑤ RegionalStim 카테터를 위쪽으로 5cm 올려 위치시킨다
⑥ 원하는 위치에 RegionalStim 카테터가 거치되면 전기자극기와 내장된 가이드와이어를 커넥터를 통해 연결한 후 낮은 자극에서 시작하여 환자가 자극을 느끼기 시작하는 자극 강도까지 올린다. 이때 최초로 자극이 느껴지는 자극의 강도를 기록하고, 자극을 느끼는 부위를 상세히 확인하여 기술한다. 이를 통해 적절한 자극의 위치를 확인한다.
⑦ 모든 환자는 경막외 마취를 시행한다. 적절한 국소마취제의 농도 및 용량을 투입하고 10분 뒤 motor, sensory block 여부 및 강도를 확인하고 분만 가능할 정도의 마취에 도달하면 분만을 진행한다.

13. 관찰항목, 임상검사항목 및 관찰검사방법

1) Primary endpoint

기존 LOR technique 방법과 RegionalStim®을 통한 방법에서의 경막외 마취 성공률 및 합병증

2) Secondary endpoint

경막외 마취 후 sensory, motor block 여부 및 강도, 마취의 적절성
경막외 마취를 시행하는데 걸린 시간, 시술 용이성
경막외 마취를 시행하는데 걸린 시간, 환자 및 시술의사의 만족도

14. 예측되는 부작용 및 사용 시 주의사항

기존 시행되던 경막외 마취로 생길 수 있는 부작용들 외에는 크게 없다(아래 기술하기로 함).

1) 경막외 마취 도중에 생길 수 있는 부작용은 소독액, 국소마취제 등으로 인한 알레르기 반응이 일어날 수 있습니다. 아주 드물게 시술 중 국소마취제 등의 약제가 의도하지 않게 혈관으로 유입될 수 있으며, 이 경우 신경독성 반응(현기증, 구토, 경련 등), 신경손상, 신경마비 등의 증상이 발생할 수 있습니다. 또한 의도하지 않게 경막천자가 일어날 수 있으며 이 경우 두통이 생길 수 있는데 증상이 심할 경우 경막외 혈액봉합술이 필요할 수도 있습니다.

2) 경막외 마취 이후 생길 수 있는 부작용은 드물지만 주사부위 경막외 출혈로 신경 주변에 혈종이 생기거나, 혹은 감염 발생할 경우 신경을 압박하거나 자극할 수 있으며 이 경우 이상감각, 신경손상, 신경마비 등의 증상이 일어날 수 있습니다. 증상이 아주 심한 경우 외과적인 치료가 필요할 수도 있습니다.

15. 연구 중지 및 탈락기준

1) 피험자가 임상시험 참여동의를 철회한 경우
2) 심각한 이상반응이나 갑작스러운 사고로 인하여 시험을 계속할 수 없는 경우

3) 피험자가 계획서에 명시된 방문 및 절차를 준수하지 않으려 하거나 할 수 없는 경우
4) 중증의 합병증의 발현으로 시험지속이 곤란한 경우
5) 선정기준에 적합하지 않은 피험자가 시험에 참여한 경우
6) 제외기준에 해당되는 피험자가 시험에 참여한 경우
7) 기타, 시험을 계속하는데 지장을 준다고 임상시험 담당자가 판단한 경우

16. 유효성의 평가기준, 평가방법 및 해석방법(통계분석방법)

1) 일차 유효성 평가변수, 이차 유효성 평가변수

① 일차 유효성 평가변수
; 기존 LOR technique 방법과 전기자극을 통한 방법에서의 경막외 마취 성공률 및 실패율

① 이차 유효성 평가변수
; 경막외 마취 후 sensory, motor block 여부 및 강도, 마취의 적절성
경막외 마취를 시행하는데 걸린 시간, 시술 용이성
경막외 마취를 시행하는데 걸린 시간, 환자 및 시술의사의 만족도

2) 유효성 평가방법 및 해석방법

현재의 LOR 방법으로만 시행한 경막외 카테터 거치 시 그 정확성을 확인할 수 있는 방법이 제한 되어 있어서 본 연구에서는 RegionalStim® 을 이용한 전기자극을 통해 경막외 공간을 확인하고 이를 기존 LOR 방법과 비교하고자 합니다. 일차 유효성 평가변수는 기존 LOR 방법과 전기자극을 통해 경막외 공간을 확인하는 방법 중 어느 것이 더 정확히 경막외 공간을 확인할 수 있는지 입니다. 두 방법으로 시행한 경막외 마취의 성공율 및 실패율을 알아보고, 추가적으로 카테터 위치에 따라 마취 강도 및 통증 조절 정도의 차이를 확인하고자 합니다. 카테터 삽입 시 전기 자극을 주었을 때 발생한 자극의 위치와 카테터를 통해 경막외 마취를 했을 때 motor, sensory block 위치 및 정도를 비교하고, 분만 중 적절한 마취가 시행되었는지, 추후 분만 후 통증 조절 시 효과에 대해서도 비교하고자 합니다.

17. 부작용을 포함한 안전성의 평가기준, 평가방법 및 보고방법

본 연구에서 발생할 수 있는 부작용은 대부분 경막외 마취의 부작용이며, 그 외의 부작용 발생가능성은 거의 없습니다. 경막외 마취는 무통분만 시 부위마취의 한 방법으로 널리 시행되고 있으며 이미 부작용 발생 시 처치 방법 및 치료에 대해 대비가 되어있는 마취 방법입니다. 발생한 부작용에 대해서는 증례기록지를 통해 기록할 것이며, 심각한 부작용이 발생할 경우 부작용 보고를 시행함은 물론 피해자 치료 및 보상에도 최선을 다할 것입니다.

18. 연구일정표

	1월	2월	3월	4월	5월	6월	7월	8월	9월	10월	11월	12월
Pilot study		⦿	⦿	⦿	⦿	⦿						
피험자 표본수 산출		⦿	⦿	⦿	⦿	⦿						
피험자 대상 임상시험			⦿	⦿	⦿	⦿	⦿	⦿	⦿	⦿		
자료 수집 및 통계 분석										⦿	⦿	
결과 분석 및 논문 작성										⦿	⦿	⦿
임상시험 결과 보고												⦿

19. 임상시험 후 피험자의 진료에 관한 사항

임상시험 후 환자는 임상시험에 참여하지 않은 일반적인 자연분만 환자와 마찬가지로 분만 후 활력징후 및 부작용에 대한 관찰을 시행할 것이며 추후 통증 관리도 필요하다면 시행할 것입니다.

20. 피험자의 안전보호에 관한 대책

본 연구는 임상시험 윤리심의위원회에서 본 연구의 윤리적, 법적 요건을 충분히 검토하여 승인한 임상시험계획서에 의하여 시행되며, 또한 시험의 전 과정에 걸쳐 임상시험 관리 기준 및 임상연구에서 의사들의 지침인 헬싱키 선언의 근본정신을 준수하게 될 것입니다. 본 연구 도중에 피험자에 인권에 침해가 발생했을 경우 임상시험 윤리심의위원회에 통보될 것입니다.

피험자의 신원을 파악할 수 있는 정보는 연구자에 의해 비밀로 보장될 것이며, 이니셜과 코드화된 피험자식별정보로 연구 자료가 기록될 것이다. 또한 피험자 및 연구정보는 접근 제한된 컴퓨터에 저장될 것이며 피험자의 신상을 보호하기 위해 독립된 공간에서 연구에 대해 설명하고 동의서를 취득할 것이다. 임상시험의 결과가 출판될 경우에도 신상정보는 비밀상태로 유지될 것입니다.

21. 그 밖에 임상시험을 안전하게 과학적으로 실시하기 위하여 필요한 사항

해당사항 없음

22. 참고문헌

1. Davies SJ, Paech MJ, Welch H et al. Maternal experience during epidural or combined spinal-epiduaral anestehsia for cesarean section: A prospective and randomized trial anesth analg 1997;85:607-613
2. Alahuhta S, Kangas-Saarela T, Hollmen AI, Edstrom HH. Visceral pain during caesarean section under spinal and epidural anesthesia with bupivacaine. Act anaesthesiologica Scandivavica 1990;34:95-98
3. Rawal N, Schollin J, Wesstrom G. Epidural versus spinal combined epidural block for cesarean section. Act anaesthesiologica Scandivavica 1988;62:31-66
4. Tsui BC, Tarkkila P, Gupta S, Kearney R. Confirmation of caudal needle placement using nerve stimulation. Anesthesiology 1999;91:374-378.
5. Tsui BC, Gupta S, Finucane B. Confirmation of epidural catheter placement using nerve stimulation. Canadian journal of anaesthesia = Journal canadien d'anesthesie 1998;45:640-644.

23. 피험자 동의서 및 설명문 양식(별첨)

첨부하였습니다.

24. 피해자 보상에 대한 규약(별첨)

첨부하였습니다.

25. 증례기록서(CRF) 양식(별첨)

첨부하였습니다.
