# Supplementary material for: Electric stimulation-guided epidural analgesia for vaginal delivery: A randomized prospective study
Source: PLoS One. 2019 Jan 11;14(1):e0209967. doi: 10.1371/journal.pone.0209967 (PMC6329494; doi:10.1371/journal.pone.0209967)
Supplement: S6 File — (PDF) [file pone.0209967.s006.pdf]

ClinicalTrials.gov PRS DRAFT Receipt (Working Version)
Last Update: 05/16/2017 21:39

ClinicalTrials.gov ID: NCT03161717

Study Identification

Unique Protocol ID: MD14043 (RegionalStimⓡ)

Brief Title: Efficacy and Safety of Electrical Stimulation-guided Epidural Analgesia for Vaginal Delivery

Official Title: A Comparative Study of Success Rate, Efficacy, Safety Between Electrical Stimulation-guided Epidural Catheter Placement and the Loss of Resistance Conventional Method for Vaginal Delivery

Secondary IDs:

Study Status

Record Verification: May 2017

Overall Status: Recruiting

Study Start: March 11, 2015 [Actual]

Primary Completion: March 2019 [Anticipated]

Study Completion: March 2019 [Anticipated]

Sponsor/Collaborators

Sponsor: Sang Sik Choi

Responsible Party: Sponsor-Investigator
Investigator: Sang Sik Choi [sschoi]
Official Title: Professor
Affiliation: Korea University Guro Hospital

Collaborators: Sewoon Medical Co., Ltd

Oversight

U.S. FDA-regulated Drug: No

U.S. FDA-regulated Device: No

U.S. FDA IND/IDE: No

Human Subjects Review: Board Status: Approved
Approval Number: MD14043
Board Name: Korea University Guro Hospital
Board Affiliation: Medical Device Institutional Review Board
Phone: 82-2-2626-1858
Email: kumdirb@gmail.com
Address:

Korea University Guro Hospital, 148, Gurodong-ro, Guro-gu, Seoul 08308, Korea

Data Monitoring: No

FDA Regulated Intervention: No

Study Description

Brief Summary: Forty pregnant women (36 to 41 weeks gestation) will randomly allocate to two groups. Groups will be defined based on the method used to identify the epidural space for epidural anesthesia: the loss of resistance group (n=20) and the epidural electrical stimulation group (n=20). Pain will be assessed using a numerical visual analog scale and maternal satisfaction by a post-partum interview. The success rate of epidural analgesia, maternal satisfaction, and neonatal Apgar scores will be compared between groups.

Detailed Description: Investigators will place epidural catheter in the epidural space using loss of resistance technique, and will confirm correct placement of the epidural catheter using electrical stimulation.

Epidural catheter placement, electrical stimulation, and confirmation of response is followed:

Patients will be placed in the left lateral decubitus position. The site will be aseptically prepared and 1% lidocaine will be infiltrated to the skin. An 18-gauge Tuohy needle will be inserted midline of L4/5 interspinous space.

For the Loss of resistance (LOR) group, after identification of the epidural space, the Tuohy needle will be stopped, and a 20-gauge epidural catheter will be advanced through the Tuohy needle.

The same process will be followed for the Epidural electrical stimulation (EES) group. In addition, the epidural space will be confirmed by epidural electrical stimulation using a 20-gauge epidural catheter (RegionalStimTm, Sewoon Medical Co., Ltd, Seoul, Korea, 800 mm) with a conductive guidewire (conductive guidewire, Nitinol, 1100 mm).

After confirming there is no reverse flow of cerebrospinal fluid or blood with aspiration, 3 mL of 1% lidocaine, with 15 mcg of epinephrine (1:200000), will be injected through the epidural catheter as test dose. If there is no response to the test dose, patients will be moved to the delivery room. To control labor pain, a one-time injection containing 50 mcg of fentanyl, 3 mL of 0.75% ropivacaine, and 6 mL of normal saline (total volume 10 mL) will be administered. A continuous infusion of 3 to 10 mL/hour depending on the patient’s pain will be used of 75 mcg of fentanyl, 8.5 mL of 0.75% ropivacaine, and 40 mL of normal saline (total volume 50 mL).

Blood pressure (BP), heart rate (HR), oxygen saturation (SpO_2), and neurologic assessment findings will be monitored up to 72 hours after labor.

Pain relief in labor is assessed by a change in the visual analogue scale (VAS) score. A 10 point VAS, where 0 is no pain and 10 is unbearable pain, is used to assess pain during labor. The scale is assessed before epidural anesthesia and after epidural anesthesia. Differences in the VAS response we used to assess the efficacy of the epidural anesthesia in decreasing labor pain. Comparison of the change in VAS between groups is used to compare pain control of the two methods. The success of epidural analgesia is defined by sensory block, without motor block, and a decrease in pain score after adequate dosing of epidural medication. Failure of epidural analgesia is defined by a lack of sensory block and a less that 2 point difference on the VAS after adequate dosing of epidural medications.

Patient satisfaction will be evaluated by a postpartum interview. Satisfaction is graded between a score of 1-5, where 1 represent very unsatisfied and 5 represent very satisfied. Patients will indicate a score of 1 to 5.

One- and 5-minute Apgar scores will be compared to assess the effect of epidural electrical stimulation on the neonate. Additional time required for epidural electrical stimulation will be determined by the difference (in seconds) from LOR to identification of the epidural space through electrical stimulation.

Conditions

Conditions:	Epidural Analgesia
Keywords:	electrical stimulation<br>vaginal delivery

Study Design

Study Type:	Interventional
Primary Purpose:	Treatment
Study Phase:	N/A
Interventional Study Model:	Parallel Assignment<br>Parallel Assignment
Number of Arms:	2
Masking:	None (Open Label)<br>No Masking
Allocation:	Randomized
Enrollment:	40 [Anticipated]

Arms and Interventions

Arms	Assigned Interventions
Experimental: Epidural electrical stimulation (EES)<br>n=20	Device: Epidural electrical stimulation (EES)<br>Using loss of resistance technique and electrical stimulation
Active Comparator: Loss of resistance (LOR)<br>n=20	Device: Loss of resistance (LOR)<br>Using loss of resistance technique only

Outcome Measures

Primary Outcome Measure:

1. Success rate of epidural analgesia
   Evaluation parameter : Accuracy comparison between loss of resistance and epidural electrical stimulation

   [Time Frame: Up to 6 months]

Secondary Outcome Measure:

2. Maternal satisfaction
   Patient satisfaction will be evaluated by a postpartum interview. Satisfaction is graded between a score of 1-5, where 1 represent very unsatisfied and 5 represent very satisfied. Patients will indicate a score of 1 to 5

   [Time Frame: Up to 6 months]

3. Neonatal Apgar score
   Assessment of neonatal

   [Time Frame: Up to 6 months]

4. Procedure-related complications
   - Check allergy reaction of anesthetics or chlorohexidine
   - Check whether Insert of local anesthetics to intravascular or not

   [Time Frame: Up to 6 months]

5. Minimum electrical current to elicit a response in the epidural electrical stimulation group
   - Check stimulation strength that patients begin the feel for the first
   - Check the proper stimulation part of body

   [Time Frame: Up to 6 months]

6. Additional time for epidural electrical stimulation
   Determined by the difference (in seconds) from loss of resistance(LOR) to identification of the epidural space through electrical stimulation

   [Time Frame: Up to 6 months]

Eligibility

Minimum Age: 19 Years

Maximum Age:

Sex: Female

Gender Based: Yes
Maternal

Accepts Healthy Volunteers: Yes

Criteria: Inclusion Criteria:

- Patients who were at 36 to 41 weeks' gestation and admitted in labor to the university clinic for vaginal delivery were included. Patients were American Society of Anesthesiologists (ASA) physical status of I or II, and were scheduled to receive epidural analgesia

Exclusion Criteria:

- Skin infection at the injection site
- Difficult catheter placement owing to previous lumbar spinal surgery or deformity
- Presence of a hemostatic disorder or use of antiplatelet therapy
- Injection of an analgesic within the previous 12 hours
- Presence of a cardiac pacemaker

Contacts/Locations

Central Contact Person: Chung Hun Lee, MD
Telephone: 82-2-2626-3240
Email: bodlch@naver.com

Central Contact Backup: Sang Sik Choi, MD, PhD
Telephone: 82-2-2626-3238
Email: clonidine@empal.com

Study Officials: Sang Sik Choi, MD, PhD
Study Principal Investigator

Korea University Guro Hiospital

Locations: South Korea
Korea University Guro Hospital
[Recruiting]
Seoul, South Korea, 08308
Contact: Seung-hoe Song, MBE 82-2-2626-1635 ssessong@korea.ac.kr
Contact: Da-in Lee 82-2-2626-2279 0124dain@gmail.com
Principal Investigator: Sang Sik Choi, MD, PhD
Sub-Investigator: Chung Hun Lee, MD
Sub-Investigator: Mi Kyoung Lee, MD, PhD

IPDSharing

Plan to Share IPD: No

References

Citations: Silva M, Halpern SH. Epidural analgesia for labor: Current techniques. Local Reg Anesth. 2010;3:143-53. doi: 10.2147/LRA.S10237. Epub 2010 Dec 8. PubMed 23144567

Wantman A, Hancox N, Howell PR. Techniques for identifying the epidural space: a survey of practice amongst anaesthetists in the UK. Anaesthesia. 2006 Apr;61(4):370-5. PubMed 16548958

Eappen S, Blinn A, Segal S. Incidence of epidural catheter replacement in parturients: a retrospective chart review. Int J Obstet Anesth. 1998 Oct;7(4):220-5. PubMed 15321183

Hermanides J, Hollmann MW, Stevens MF, Lirk P. Failed epidural: causes and management. Br J Anaesth. 2012 Aug;109(2):144-54. doi: 10.1093/bja/aes214. Epub 2012 Jun 26. Review. PubMed 22735301

Tsui BC, Tarkkila P, Gupta S, Kearney R. Confirmation of caudal needle placement using nerve stimulation. Anesthesiology. 1999 Aug;91(2):374-8. PubMed 10443599

Tsui BC, Gupta S, Finucane B. Confirmation of epidural catheter placement using nerve stimulation. Can J Anaesth. 1998 Jul;45(7):640-4. PubMed 9717595

Links:

Available IPD/Information:
